# Assessment of life loss due to dam breach using improved variable fuzzy method

**DOI:** 10.1038/s41598-022-07136-0

**Published:** 2022-02-25

**Authors:** Hongbo Jiao, Wei Li, Ding Ma

**Affiliations:** 1 Organization Department, North China University of Water Resources and Electric Power, Zhengzhou, 450001 China; 2 School of Railway Engineering, Zhengzhou Railway Vocational and Technical College, Zhengzhou, 450001 China; 3 Limkokwing University of Creative Technology, Cyberjaya, Malaysia

**Keywords:** Hydrology, Natural hazards

## Abstract

In recent years, several factors, such as frequent extreme weather, disrepair of dams, and improper management, have caused frequent dam failures, posing a significant threat to people's lives downstream. At present, the life loss is evaluated using the empirical formula method, in which the recommended approximate and threshold results are obtained through linear regression or statistical analysis. However, this method is sometimes insufficient because of the lack of a historical dataset and low availability, and it tends to simplify or ignore the influence of some factors in regression. During the research, most objects are considered as individual cases, and thus, the universality and scientificity of the application of evaluation models or parameters need further discussion. The variable fuzzy set theory features rigorous mathematical clarity and fuzziness of things and is widely used in the optimal decision evaluation model. Although, the traditional variable fuzzy evaluation method is widely used to deal with the linear variation in the index, some indexes, such as dam storage capacity and downstream population at risk, can cause non-linear problems, directly affecting the accuracy of membership evaluation results. Therefore, an improved model was proposed, where the relative difference formula was improved through logarithmic transformation and boundary constraint. The improved method was applied to the sequencing of life loss risk consequences for four reservoirs. The evaluation result was consistent with the actual situation of the disaster and the actual mortality rate. The scientificity and practicability of the improved model were verified, providing a new perspective for reservoir risk ranking and enriching the risk management theory.

## Introduction

China has a huge number of reservoir dams, and several reservoirs have problems such as large seepage, insufficient flood control, and inadequate management. Dam outburst significantly impacts the downstream, and loss of life is an important factor for the evaluation of dam risk consequences. The evaluation of the risk of life loss caused by dam breaks remains a key technical problem in the research on dam risk management in China and abroad^1^. At present, the domestic and overseas studies on life loss use an empirical formula method, in which the recommended approximate and threshold results are obtained through linear regression or statistical analysis^2–12^. However, this method significantly depends on the database, and thus, it tends to simplify or ignore the influence of some factors in the regression. During the research, most objects are considered as special cases, thus, the universality and scientificity of the application of the evaluation model or parameters need further discussion^13–15^. The lack of historical data and references in risk identification and evaluation limits the application of the empirical formula method in loss-of-life research^16^.

Chinese researcher Chen Shou^17–19^ proposed the variable fuzzy set theory based on the relative difference function for the first time. The variable fuzzy set theory uses rigorous mathematics unifying the clarity and fuzziness of things, and it is widely used in the optimal decision evaluation model^20–25^. However, the traditional variable fuzzy evaluation method is widely used to deal with the linear variation of the index. The relative difference degree of common evaluation indicators in life loss assessment, such as storage capacity and at-risk population, causes non-linear problems, thus directly affecting the accuracy of membership evaluation results. Therefore, it is theoretically and practically significant to improve the variable fuzzy evaluation model and apply it to the evaluation of the life loss in a dam break.

## Methods

### Basic theory of variable fuzzy evaluation method

The relative difference degree is the core of variable fuzzy theory^26,27^, characterizing the dynamic features of the intermediary transition of the fuzzy concept by describing the attractability and repellency of things. It is not limited by Zade’s static fuzzy set and marks the entry of the traditional fuzzy mathematical theory into the theory of variable fuzzy sets. The relative difference function aims to study the clarity fuzziness of objective things during changes and lays the theoretical foundation of the variable fuzzy evaluation method.

### Relative difference degree

We consider the opposing fuzzy concept on domain U, u is an element in U, and u $\in$. At any point axis in the continuous system of the relative membership function, the relative membership degree of u to $\hat{A}$, which represents the attractability, is $\mu_{{\hat{A}}} \left( u \right)$, and that to $\widehat{{A_{c} }}$, which represents the repellency, is $\mu_{{\hat{A}_{c} }} \left( u \right)$; $\mu_{{\hat{A}}} \left( u \right)$[0,1] and $\mu_{{\hat{A}_{c} }} \left( u \right)$[0,1]. Thus, $D_{{\hat{A}}} \left( u \right)$ is called the relative difference degree of U to Â, as shown in Eq. (1).

1 $$D_{{\hat{A}}} \left( u \right) = \mu_{{\hat{A}}} \left( u \right) - \mu_{{\hat{A}_{c} }} \left( u \right)$$

Because of

2 $$\mu_{{\hat{A}}} \left( u \right) + \mu_{{\hat{A}_{c} }} \left( u \right) = 1$$

Then the relative difference degree of u to $\hat{A}$ is shown in formula (3).

3 $$\mu_{{\hat{A}}} \left( u \right) = \left( {1 + D_{{\hat{A}}} \left( u \right)} \right)/2$$

### Relative difference function model

**Figure 1:**
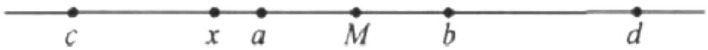
Location diagram of X0,X.

Let X0 = [a,b] be the attraction domain of the fuzzy variable set $\hat{\user2{V}}$ on the real axis and X = [c,d] be the upper and lower range interval domains that contain X0, as shown in Fig. 1.

Let *M* be the point value of $D_{{\hat{A}}} \left( u \right) = 1$ in the attraction domain interval [a,b] and X be the measure of any point in the X interval; thus, the relative difference function model of *x* falling to the left of point *M* is expressed as Eqs. (4) and (5).

4 $$D_{{\hat{A}}} \left( u \right) = \left( {\frac{x - a}{{M - a}}} \right)^{\beta } ,x \in \left[ {a,M} \right]$$

5 $$D_{{\hat{A}}} \left( u \right) = \left( {\frac{x - a}{{M - a}}} \right)^{\beta } ,x \in \left[ {a,M} \right]$$

when x falls to the right of point M, its relative difference function model is expressed as Eqs. (6) and (7).

6 $$D_{{\hat{A}}} \left( u \right) = \left( {\frac{x - b}{{M - b}}} \right)^{\beta } ,x \in \left[ {M,b} \right]$$

7 $$D_{{\hat{A}}} \left( u \right) = - \left( {\frac{x - b}{{d - b}}} \right)^{\beta } ,x \in \left[ {b,d} \right]$$

In the equation, $\beta$ is a non-negative exponent, usually considered as $\beta$ = 1, that is, the relative difference function is a linear model.

The variable fuzzy evaluation method and the given relative difference function can quantify the difference degree of the index relative to its standard value interval at all levels. Thus, it can determine the membership degree of the index standard value relative to the interval, providing a new way of performing the multi-index and multi-level comprehensive evaluation under the condition that the standard value is the interval.

### Improvement of variable fuzzy membership function

From the derivation process of the relative difference degree and membership formula, it can be seen that there is a complex functional relationship of the determined membership μ with the index value x, interval boundary value b, and intermediate point value M. When the index is within a particular attraction domain range, the evaluation object has an absolute affiliation with the interval. As the indicator changes, and the indicator is within the adjacent exclusion domain interval, the affiliation decreases. When the indicator reaches the median value of the adjacent interval, the affiliation disappears. Because of the characteristics of the intermediary transition, there is an equilibrium at the interval boundary. At this time, the membership relationship is no longer absolute, but remains relatively neutral. The membership function should be smooth during the transition, and the convergence acceleration changes to a certain value. In addition, the membership function should converge linearly, that is, there should be an approximate linear correlation between the changes in the index and membership degree. However, factors affecting the risk of life loss in dam breaks, such as the features of the reservoir, risk population, and time of the dam break, are unclear or show exponential changes, as shown in Table 1.

**Table 1:** Risk index and evaluation standard of life loss in dam break flood.

First indicator	Second indicator	Notion	Level1	Level2	Level3	Level4	Level5
			Alight	Normal	Medium	Serious	Extremely serious
Dangerous indicator X_1_	X_11_	Capacity C_R_/(10^5^ × m^3^)	1–10	10–102	102–103	103–104	> 104
	X_12_	Flood intensity S_F_/(m^2^/s)	0–0.5	0.5–4.6	4.6–12	12–15	> 15
Exposure indicator X_2_	X_21_	Distance L_D_/(km)	> 50	50–20	20–10	10–5	5–0
	X_22_	Time T_B_	0–1	1–10	10–102	102–103	103–104
Delicate indicator X_3_	X_31_	Risk population P_R_/(person)	1–102	102–103	103–104	104–105	> 105
	X_32_	Understanding of flood U_B_	Very clear	Clear	Normal	Vogue	Extremely vogue

According to Eqs. (6) and (7), because d is the exponential difference of b, which is much different from d and M, the use of the traditional function model leads to a significant jump when the membership degree maps to the indicator x on both sides of b. The jump in the results of the membership calculation affects the accuracy of the evaluation results. Therefore, based on the limitations of the application, the following improvement methods were proposed in this paper.

#### Improvement of relative membership degree model

The core of the variable fuzzy evaluation method calculation is the relative difference degree and relative membership degree. Assuming that $x_{ij}$, the characteristic indicator of the sample $u_{j}$, falls into [$M_{ih}$, $M_{{i\left( {h + 1} \right)}}$], which is the matrix M of the adjacent levels h and (h + 1), and when the segment of the indicator level shows a linear change, the relative membership degree of the indicator i to level h can be expressed as Eq. (8).

8 $$\left\{ {\begin{array}{*{20}c} {\mu_{ih} \left( {u_{j} } \right) = 0.5\left( {1 + \frac{{b_{ih} - x_{ij} }}{{b_{ih} - M_{ih} }}} \right),x_{ij} \in \left[ {M_{ih} ,b_{ih} } \right]} \\ {\mu_{ih} \left( {u_{j} } \right) = 0.5\left( {1 - \frac{{b_{ih} - x_{ij} }}{{b_{ih} - M_{{i\left( {h + 1} \right)}} }}} \right),x_{ij} \in \left[ {b_{ih} ,M_{{i\left( {h + 1} \right)}} } \right]} \\ \end{array} } \right.,h = 1,2, \ldots ,n$$

when the segmentation of the indicator level exhibits a nonlinear change, the relative membership degree of the indicator i to level h should also change with a nonlinearity. For the index of the index level change in life loss evaluation, the calculation of its membership degree should be a logarithmic conversion processing to synchronize the corresponding change. The relative membership calculation formula in this case is expressed as Eq. (9).

9 $$\left\{ {\begin{array}{*{20}c} {\mu_{ih} \left( {u_{j} } \right) = 0.5\left( {1 + D_{1} \left( x \right)} \right),x_{ij} \in \left[ {M_{ih} ,b_{ih} } \right]} \\ {\mu_{ih} \left( {u_{j} } \right) = 0.5\left( {1 + D_{2} \left( x \right)} \right),x_{ij} \in \left[ {b_{ih} ,M_{{i\left( {h + 1} \right)}} } \right]} \\ \end{array} } \right.$$

The relative difference degree function $D_{1} \left( x \right)$ should be a monotonically decreasing concave function, thus:

10 $$D_{1} \left( x \right) = \log_{\lambda } \frac{{C_{1} }}{{x + C_{2} }}$$

The constraint condition is expressed as Eq. (11).

11 $$\left\{ {\begin{array}{*{20}c} {D_{1} \left( {M_{ih} } \right) = 1} \\ {D_{1} \left( {b_{ih} } \right) = 0} \\ \end{array} } \right.D_{1} \left( x \right) = \log_{\lambda } \frac{{C_{1} }}{{x + C_{2} }}$$

The relative difference degree function $D_{1} \left( x \right)$, which satisfies the above constraints, is expressed as Eq. (12).

12 $$D_{1} \left( x \right) = \log_{\lambda } \frac{{\lambda \left( {b_{ih} - M_{ih} } \right)}}{{\left( {\lambda - 1} \right)x - \lambda M_{ih} + b_{ih} }}$$

Similarly, the relative difference degree function $D_{2} \left( x \right)$ can be expressed as Eq. (13).

13 $$D_{2} \left( x \right) = \log_{\lambda } \frac{{M_{{i\left( {h + 1} \right)}} - b_{ih} }}{{\left( {\lambda - 1} \right)x - \lambda b_{ih} + M_{{i\left( {h + 1} \right)}} }}$$

Substituting the formula of the functions $D_{1} \left( x \right)$ and ${ }D_{2} \left( x \right)$ into Eq. (9), the improved relative membership formula can be expressed as Eq. (14).

14 $$\left\{ {\begin{array}{*{20}c} {\mu_{ih} \left( {u_{j} } \right) = 0.5\left( {1 + \log_{\lambda } \frac{{\lambda \left( {b_{ih} - M_{ih} } \right)}}{{\left( {\lambda - 1} \right)x - \lambda M_{ih} + b_{ih} }}} \right),x_{ij} \in \left[ {M_{ih} ,b_{ih} } \right]} \\ {\mu_{ih} \left( {u_{j} } \right) = 0.5\left( {1 + \log_{\lambda } \frac{{M_{{i\left( {h + 1} \right)}} - b_{ih} }}{{\left( {\lambda - 1} \right)x - \lambda b_{ih} + M_{{i\left( {h + 1} \right)}} }}} \right),x_{ij} \in \left[ {b_{ih} ,M_{{i\left( {h + 1} \right)}} } \right]} \\ \end{array} } \right.$$

The relative membership degree of indicator x, which is smaller than h and greater than h + 1, should be 0, i.e., $\mu_{{i\left( { < h} \right)}} \left( {u_{j} } \right) = 0,\mu_{{i\left( { > \left( {h + 1} \right)} \right)}} \left( {u_{j} } \right) = 0$. When x_ij_ is outside the upper and lower bound ranges, μ_i1(u_j) = μ_in(u_j) = 1.

#### Rationality verification of the improved method

As a statistical index^28^ reflecting the close correlation between variables, the correlation coefficient represents the correlation between the two variables by multiplying two deviations based on the deviation between the two variables and their respective averages. The Pearson correlation coefficients are widely applied to measure the correlation between two dataset distance variables. The closer the coefficients are to 1 or − 1, the stronger the correlation; the closer they are to 0, the weaker the correlation. The correlation coefficient is represented by r; the correlation coefficient formula of the variable indicator *x* and membership *μ* is expressed as Eq. (15).

15 $$r\left( {x,\mu } \right) = \frac{{Cov\left( {x,\mu } \right)}}{{\sqrt {Var\left[ x \right]Var\left[ \mu \right]} }}$$

The attraction domain [a,b] and the upper and lower boundary range domains [c,d] of the fuzzy variable set are assumed to be [100,1000] and [10,10000] respectively, which is the case of a typical exponential level distribution index. A membership output of 1000 and its indexes before and after improvement are obtained; similarly, the correlation coefficient of the two variables are also obtained, as summarized in Table 2.

**Table 2:** Comparison of correlation coefficients of indication example and membership degree before and after improvement.

Indicator value X	800	850	900	950	1000	1050	1100	1150	1200	Correlation coefficient R
Affiliate degree *µ* (after improved)	0.567	0.548	0.531	0.515	0.5	0.479	0.46	0.443	0.427	0.999
Affiliate degree *µ* (before improved)	0.648	0.611	0.574	0.537	0.5	0.494	0.489	0.483	0.478	0.876

The result in the above table suggests that the membership degree calculated by the traditional variable fuzzy model lies on both sides of the attraction domain interval boundary point, and its changing trend appears to be leaping. For example, on the left side of 1000, the index changes by approximately 0.037 per 50 of the indicator value. For the same indicator change on the right side of 1000, the membership change is 0.005, showing a difference of 7.4 times, directly affecting the accuracy of the calculation result at the risk evaluation level. Meanwhile, the improved model output presents a better linear correlation than before and reflects the mapping relationship between the index and membership degree more scientifically.

## Results and discussion

### Calculation process

**Figure 2:**
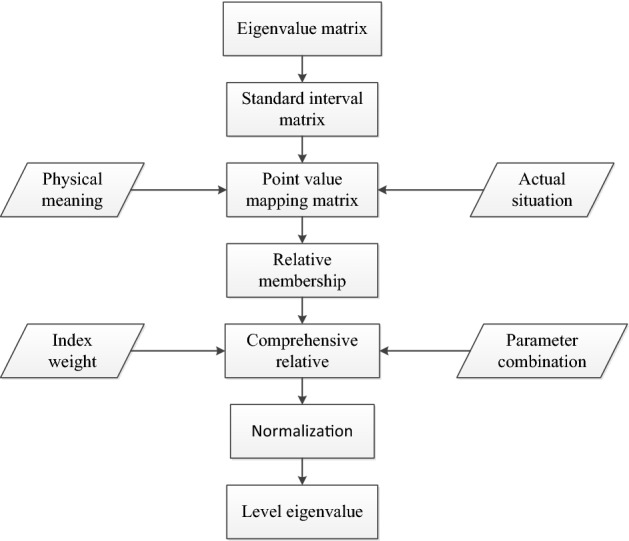
Variable fuzzy evaluation steps.

Based on the aforementioned research, the calculation process of improving the risk evaluation method of life loss in a variable fuzzy dam break is shown in Fig. 2.

The specific calculation steps are as follows: (1) The sample eigenvalue matrix is determined according to the characteristic value of the reservoir index to be evaluated; (2) The standard interval matrix of the index is obtained based on the index classification interval; (3) The standard interval point value mapping matrix is determined in accordance with the physical meaning and actual situation; (4) The relative membership matrix of the indicator *x*_*ij*_ at all levels is determined; (5) The parameter combination of *α*, *p* is changed, and Eq. (16) is used to calculate the comprehensive relative membership degree; (6) Normalization of the comprehensive relative membership vector is followed by the calculation of the risk level eigenvalue of the evaluation sample using Eq. (16); (7) The sample risk level is evaluated according to the level eigenvalue.

16 $$v_{h} \left( {u_{j} } \right) = \frac{1}{{1 + \left\{ {\frac{{\mathop \sum \nolimits_{i = 1}^{m} \left[ {\omega_{i} \left( {1 - \mu_{ih} } \right)} \right]^{p} }}{{\mathop \sum \nolimits_{i = 1}^{m} \left[ {\omega_{i} \cdot \mu_{ih} } \right]^{p} }}} \right\}^{{\frac{\alpha }{p}}} }}$$

In Eq. (16), $\omega_{i}$ is the weight coefficient^29^ of the indicator i satisfying $0 < \omega_{i} < 1,\mathop \sum \limits_{i = 1}^{m} \omega_{i} = 1.{\text{ The }}\alpha$ represents the optimized criteria parameters, α = 1 is the least absolute criterion, and α = 2 is the least square criterion; p is the distance parameter, *p* = 1 is the hemming distance, and *p* = 2 is the Euclidean distance.

In the Equation, $v_{h}^{^{\prime}}$ is the normalized comprehensive relative membership degree, and *H* is the level eigenvalue of the evaluation sample.

17 $$H = \mathop \sum \limits_{h = 1}^{n} v_{h}^{^{\prime}} \cdot h$$

### Subjects of study

Four broken dams in China^30-32^ were selected as the evaluation objects, and the value assignment for the qualitative index was based on the investigation of the dam break combined with the index grade standard. The sample reservoir profiles are presented in Table 3.

**Table 3:** Summary of investigation of four outburst dams.

Item	Outburst dams	Province	Date	Reservoir capacity	People at risk in the downstream
				(10^5^ × m^3^)	(Persons)
1	Hengjiang	Guangdong	1970-09-15	787.9	2500
2	Dongkou	Zhejiang	1971-06-02	25.5	3500
3	Lijia Tsui	Gansu	1973-04-29	11.4	1034
4	Historian Gou	Gansu	1973-08-25	8.56	300

### Model calculation and result analysis

According to the dam break investigation data and collation results, the risk level of life loss for the four reservoirs was calculated following the calculation process discussed in Sect. Calculation Process, and the scientificity and reliability of the calculation results were verified by comparing and analyzing the reality of the disaster. The calculation results are summarized in Table 4.

**Table 4:** Calculation results of risk level eigenvalue.

Sample serial number	Dam break reservoir	Grade characteristic value H				Risk level ${ }\overline{H}$	Loss of life (person)	Death rate %
		$\alpha$ = 1, *p* = 1	$\alpha$ = 1, *p* = 2	$\alpha$ = 2, *p* = 1	$\alpha$ = 2, *p* = 2			
1	Hengjiang	2.7582	2.8583	2.5143	2.6279	2.6897	20	0.8
2	Dongkou	3.1240	3.2072	2.9446	3.0211	3.0742	154	4.4
3	Lijia Tsui	3.4697	3.3585	3.7024	3.6047	3.5338	516	49.9
4	Shijia Gou	3.3703	3.2265	3.5240	3.3047	3.3564	81	27

**Figure 3:**
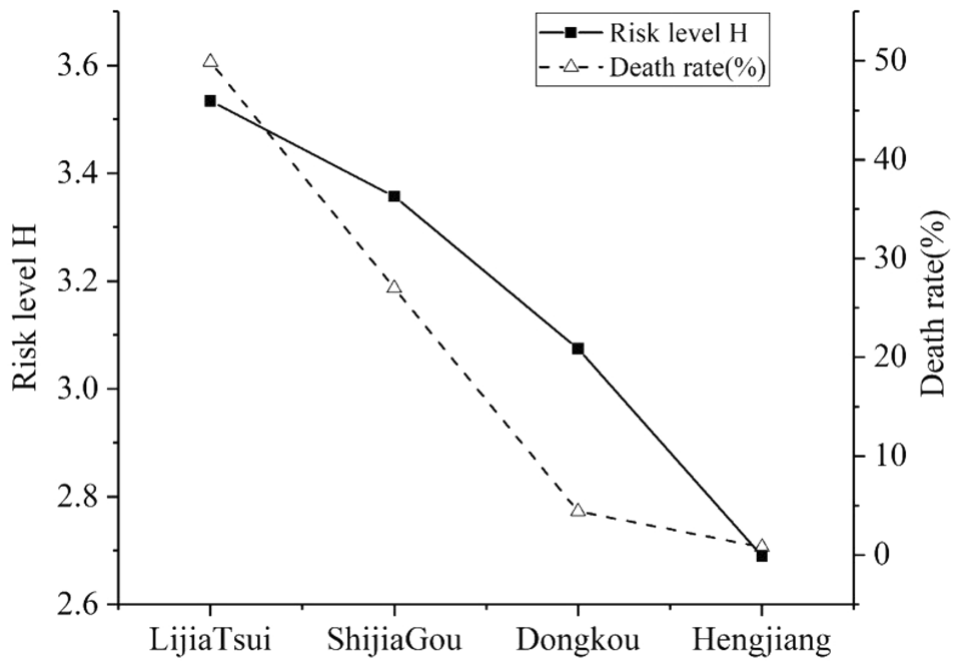
Comparison of life-loss risk levels and death rates, this figure is created by Microsoft Office 2013 (https://www.microsoftstore.com.cn/software/office).

Based on the results in Table 4, the trend chart of the eigenvalue change of the life-loss risk level for the four sample reservoirs is plotted, as shown in Fig. 3.

The risk ranking of the four reservoirs is Lijia Tsui > Shijia Gou > Dongkou > Hengjiang. Apart from the Hengjiang reservoir, which is at a medium risk level, the risk levels h̅ of the other reservoirs are in [3,4] intervals, which are severe. The actual death toll and death rate for the Hengjiang reservoir were the lowest among the four reservoirs, thus, the lowest risk level is in line with reality. The actual disaster of the Lijia Tsui reservoir had a significant impact on the people living in the downstream area, with 516 deaths and an at-risk population of 1034, and the mortality rate was nearly 50%. Nearly half of the villagers died owing to the dam-break floods, thus, it is reasonable to rank the reservoir at the top in terms of risk levels. Although the death toll for the Shijia Gou reservoir was only 81, which was lower than that for the Dongkou reservoir, as the population living downstream of the reservoir was only 300 people, the mortality rate had reached 27%, which was much higher than 4.4%, the rate for the Dongkou reservoir. Therefore, the life risk consequences of the Shijia Gou reservoir were larger, which is more consistent with people's feelings about the risk consequences of dam collapse. Meanwhile, the ranking of life-loss risk expressed by level eigenvalues is consistent with the ranking trend obtained using the mortality size. In summary, the ranking of life loss caused by dam breaks obtained through the improved model effectively reflects the severity of disaster life risk, showing the accuracy of the method in predicting the life-loss risk resulting from dam breaks.

## Conclusion

When the variable fuzzy evaluation method is used to evaluate the life loss due to a dam break, the method is vulnerable to errors caused by membership leaps. In this study, more reliable results of the variable fuzzy evaluation method was obtained by improving the relative difference degree function through the logarithmic transformation and boundary constraint methods. The risk evaluation index system for life loss resulting from dam breaks was established based on the theory of disaster system and three aspects: dangers of hazard factors, exposure to the hazard-inducing environment, and vulnerability of the hazard-bearing body. The risk ranking of the four samples was determined using the improved variable fuzzy evaluation method. The results were consistent with those of the actual disaster and mortality sequencing, which verifies the method’s scientificity and applicability in evaluating the life risk associated with dam-break disasters and provides a new perspective and scientific method for the study of the risk consequences of life loss caused by dam breaks.
